# Tanshinone IIA attenuates nerve structural and functional damage induced by nerve crush injury in rats

**DOI:** 10.1371/journal.pone.0202532

**Published:** 2018-08-23

**Authors:** Zhiyong Wang, Xiaomei Yang, Weiguang Zhang, Peixun Zhang, Baoguo Jiang

**Affiliations:** 1 Department of Anatomy and Histo-embryology, School of Basic Medical Sciences, Peking University, Beijing, China; 2 Department of Trauma and Orthopedics, Peking University People's Hospital, Beijing, China; University of Sydney, AUSTRALIA

## Abstract

After peripheral nerve crush injury, the fibers of distal nerve segments gradually disintegrate, and axons regrow from the proximal nerve segment, eventually reaching the target organ. However, the axon regeneration is generally not sufficient for the recovery of neurological function, so drug therapy is necessary. In the current study, we explored the effect of Tanshinone IIA in nerve regeneration in a sciatic nerve crush injury model using Sprague Dawley rats. The rats were administered 45 mg/kg of Tanshinone IIA once daily. Motor behavior and tibialis anterior muscle mass were assessed, and histological analysis of the sciatic nerve and lumbar spinal cord were conducted. The results showed that the administration of Tanshinone IIA improved nerve growth and motor function, and resulted in a marked decrease of neuronal death. The findings of this exploratory study suggest that Tanshinone IIA alleviates injury and boosts regeneration after nerve crush injury in a rat model of sciatic nerve injury.

## 1. Introduction

Neural function can be partially or completely lost after injury. The loss of function often results in limitations of health-related quality of life. Following nerve crush injury, the fibers of distal nerve segments gradually disintegrate, and the debris is eventually cleared by Schwann cells and macrophages. Subsequently, axon regrowth of the proximal nerve segment is induced to regenerate into the distal endoneurial tube, and eventually reinnervate the target organs. However, the axon regeneration is generally not sufficient for the recovery of neurological structure or function. To promote nerve regeneration, drug therapy is necessary.

Chinese herbal medicine is often used as adjuvant treatment for nerve injury because of few side effects. Quercetin, Achyranthes bidentata polypeptides, and angelica could improve nerve growth after nerve crush injury [1–3]. However, Chinese herbal medicines that more effectively treat nerve crush injury have yet to be discovered, still needing further exploration to excavate more related herbs. Therefore, the aim of the present study was to identify other Chinese herbal medicines that have potential to treat peripheral nerve crush injury.

We investigated Tanshinone IIA, a component of the Chinese herbal medicine Danshen [4], as a treatment following nerve crush injury. As the primary component of Danshen, Tanshinone IIA defends the brain against cerebral ischemia reperfusion damage [5], demonstrating neuroprotective capacities in the central nervous system. More importantly, Tanshinone IIA attenuates the injury in a sciatic nerve transection model, suggesting its efficacy on peripheral nerve injury [6]. On the basis of the above findings, we speculate that Tanshinone IIA has the potential for attenuating injury in a nerve crush injury model. In this study, we examined the role of Tanshinone IIA in the sciatic nerve following crush damage to determine its effect on peripheral nerve injury, and explored its application in other aspects of nerve injury.

## 2. Materials and methods

### 2.1. Ethics statement

Rats experiments adhered to the ARRIVE guidelines [7]. The experimental procedures were conducted according to the U.K. Animals Act as well as the Chinese guidelines for using laboratory animals. The study protocols of the rats were approved by the Animal Care and Use Committee of Peking University.

### 2.2. Surgical procedure and grouping

**Fig 1:**
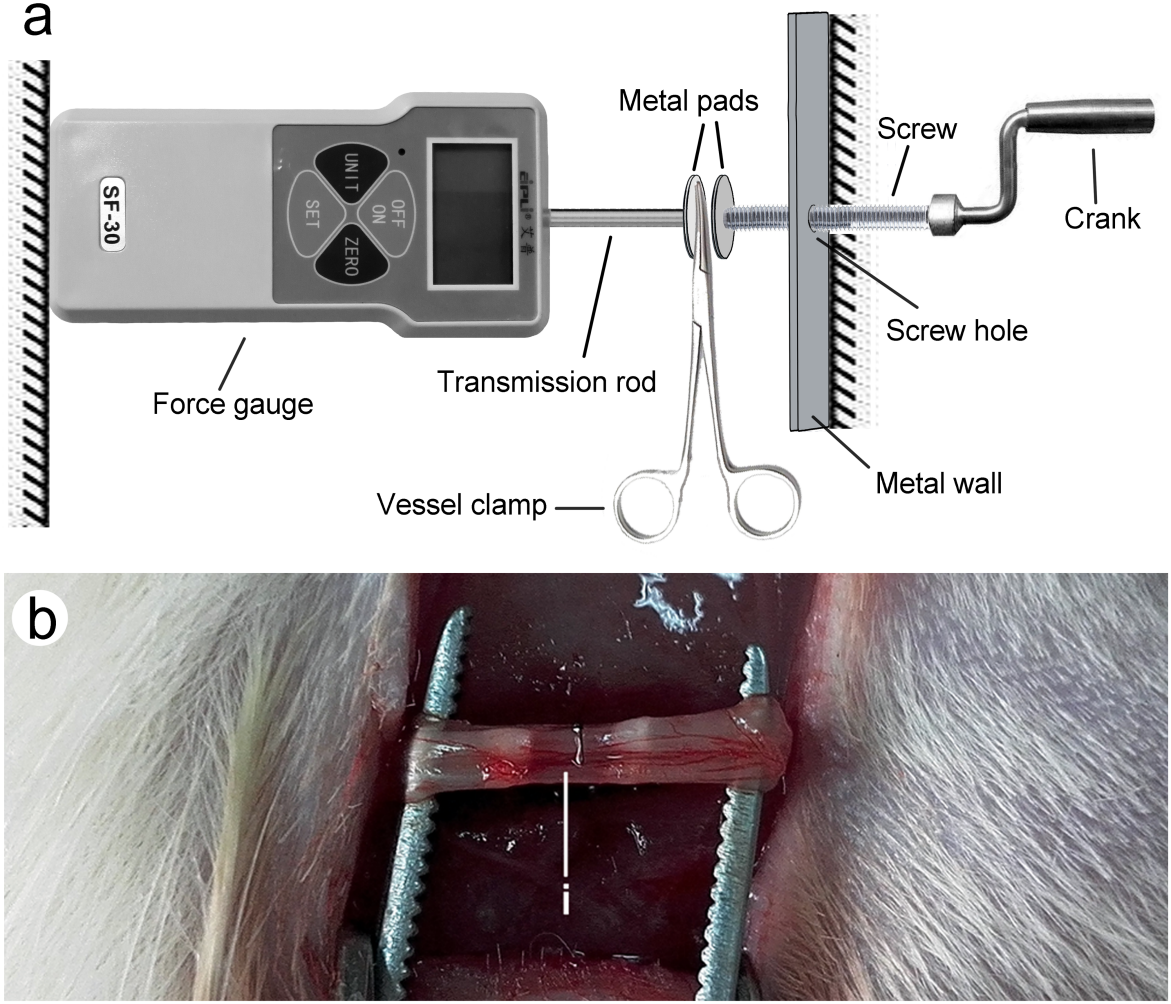
Crush injury model. (a) Method for producing the crush injury model. (b) Sciatic nerve that was crushed; (i) indicates crushing site.

Eighteen male Sprague Dawley rats (purchased from the animal center of Peking University Health Science Center where the animals were bred by Dr. Zhang for research purposes) weighing 220–240 g were housed at an animal facility under specific pathogen-free and climate-controlled conditions with a 12 h light/dark cycle. The animals were housed in polystyrene cages with wood shavings (3 rats in each cage; cage dimensions: 330 × 215 × 170 mm). The rats were provided with water and a standard diet ad libitum. Twelve rats were randomly chosen by a researcher blinded to the study design, and the rats were intraperitoneally injected with sodium pentobarbital (32 mg/kg) for anesthesia before the experiment. Another researcher who was blind to the study design monitored all of the procedures to ensure the absence of neural reflexes. The effectiveness of anesthesia was ideal when the rats are asleep without neural reflexes. Surgery was performed using aseptic techniques. The right sciatic nerves were exposed. At a site 5 mm proximal to the main bifurcation, a toothless vessel clamp was used to crush the sciatic nerve. The process was as follows. The force gauge and a metal wall with a screw hole were fixed on a table. A toothless vessel clamp slightly clamping the sciatic nerve was placed between the metal pads, allowing the sciatic nerve to contact the vascular forceps, but not be squeezed. The crank was rotated to allow the metal pads to lightly touch the vessel clamp, then the force gauge was zeroed, and the crank was further rotated until the pressure increased to 30 N. The duration of the crush was 1 min. After the operation was completed, the rats were randomly allocated to a control group and a treatment group. The number of rats in each group was 6, according to medical statistics and previous studies [8]. In an effort to reduce unnecessary animal use, we used the minimum number of rats in the allowed range. As for the sham group, the nerve was exposed, and the incision was sutured without performing the nerve crush. The body temperature and weight of rats in the different groups were measured every day to make sure that the physiological indicators of the rats were normal. All efforts were made to minimize suffering (Fig 1).

### 2.3. Drug treatment

Tanshinone IIA (Biorbyt, Wuhan) was dissolved in 0.9% sterile saline. Rats in the sham and control groups (n = 6 in each group) were administered with 0.9% sterile saline by intraperitoneal injection at 8:00 AM once daily. Rats in the treatment group (n = 6) received a 5 ml intraperitoneal injection of Tanshinone IIA (45 mg/kg) once daily at the same time every day, for a period of 4 weeks [6].

### 2.4. Behavioral analysis

Motor function analysis was conducted on the animals of all groups as previously described [8]. A self-made sealed corridor (length, width and height were 10, 10 and 70 cm, respectively) was used. White paper (10 × 70 cm) was put on the ground under the corridor. The two hind limbs were stained with red pigment, then the rat was placed at the entrance of the corridor. The bilateral footprints were recorded. The above process was repeated three times. Clear and complete prints were chosen for measurement. Footprint parameters were measured: Print length (PL), Toe spread (TS) and intermediary toe spread (IT). The parameters about left foot were recorded as NPL, NTS and NIT. The parameters about right foot were recorded as EPL, ETS and EIT. Sciatic function index (SFI) was determined by the formula: SFI = −38.3 ([EPL − NPL] / NPL) + 109.5 ([ETS − NTS] / NTS) + 13.3 ([EIT − NIT] / NIT) − 8.8.

### 2.5. Evaluation of tibialis anterior muscle mass

Tibialis anterior muscles were harvested from the both hindlimbs of the animals. To reduce statistical error and ensure objectivity, the tibialis anterior muscles used for analysis included the tendons. Muscles were then immediately weighed. The weight ratio was obtained by dividing the weight of the right muscle by the weight of the left one.

After being weighed, each triceps surae muscle was then fixed in 4% paraformaldehyde. Each tissue was then immersed in water, followed by ethanol, xylene, and finally embedded. Each segment was used for HE staining. Images were acquired under a Leica dissecting microscope.

### 2.6. Histological analysis of sciatic nerves

The sciatic nerve distal to the injury site was harvested and divided into two sections. One section was used for optical microscopy. It was fixed in paraformaldehyde for 12 h, rinsed twice in water, stained with 1% osmium tetroxide, embedded in paraffin, finally sliced into 4-μm-thick sections with a Leica tissue microtome. Slices were observed and myelinated fibers were counted.

The other section was used for transmission electron microscopy (TEM). The tissue was successively immersed in 3% glutaraldehyde, 1% osmium tetroxide, ethanol, and a mixture of epoxy resin and acetone (1:1), then finally embedded in epoxy resin. Then, ultrathin sections (50.0 nm thickness) were prepared, stained using uranyl acetate solution and lead citrate solution, and finally observed using a transmission electron microscope (JEM-100), from which morphometric evaluation was conducted, including axon diameter, myelin thickness and fiber diameter being evaluated using Image J software. The g-ratio was calculated as follows: g-ratio = axon diameter / fiber diameter.

### 2.7. Assessment of spinal cord neurons

Assessment of spinal cord neurons was performed by a researcher blinded to the animal allocation. The spinal cord at L4–L6 was harvested, fixed in 4% paraformaldehyde, and made into paraffin sections. Sections were incubated with the primary antibody anti-NeuN (1:400, Abcam, UK) at 37 degrees Celsius for 2 h. The secondary antibody (biotinylated IgG, Abcam) was added, and the sections were incubated for 20 min. The reactions were developed with 3–3’ diaminobenzidine peroxidase (ZSGB, China). Counting of anterior horn motoneurons was performed using Image J. A horizontal line was drawn through the central canal of the spinal cord, and immunopositive cells were counted in the gray matter area on the ventral side of the line.

### 2.8. Randomisation and assessment order

Animals were randomly allocated to experimental and sham groups, all tests were performed by a researcher blinded to the animal allocation. The test order was random in the different experimental groups.

### 2.9. Euthanasia

At 4 weeks after the experiment, all animals were sedated and anesthetized prior to euthanasia. The rats were then euthanized by transcardial perfusion with a 4% paraformaldehyde solution and intravenous injection with thiopental and potassium chloride.

### 2.10. Statistical analysis

Two-way analysis of variance was used to analyze SFI, and one-way analysis of variance was used to analyze all other data. Statistical significance was set at P < 0.05 for all statistical comparisons. All values are presented as the mean ± SD.

## 3. Results

### 3.1. Sciatic function index (SFI)

**Fig 2:**
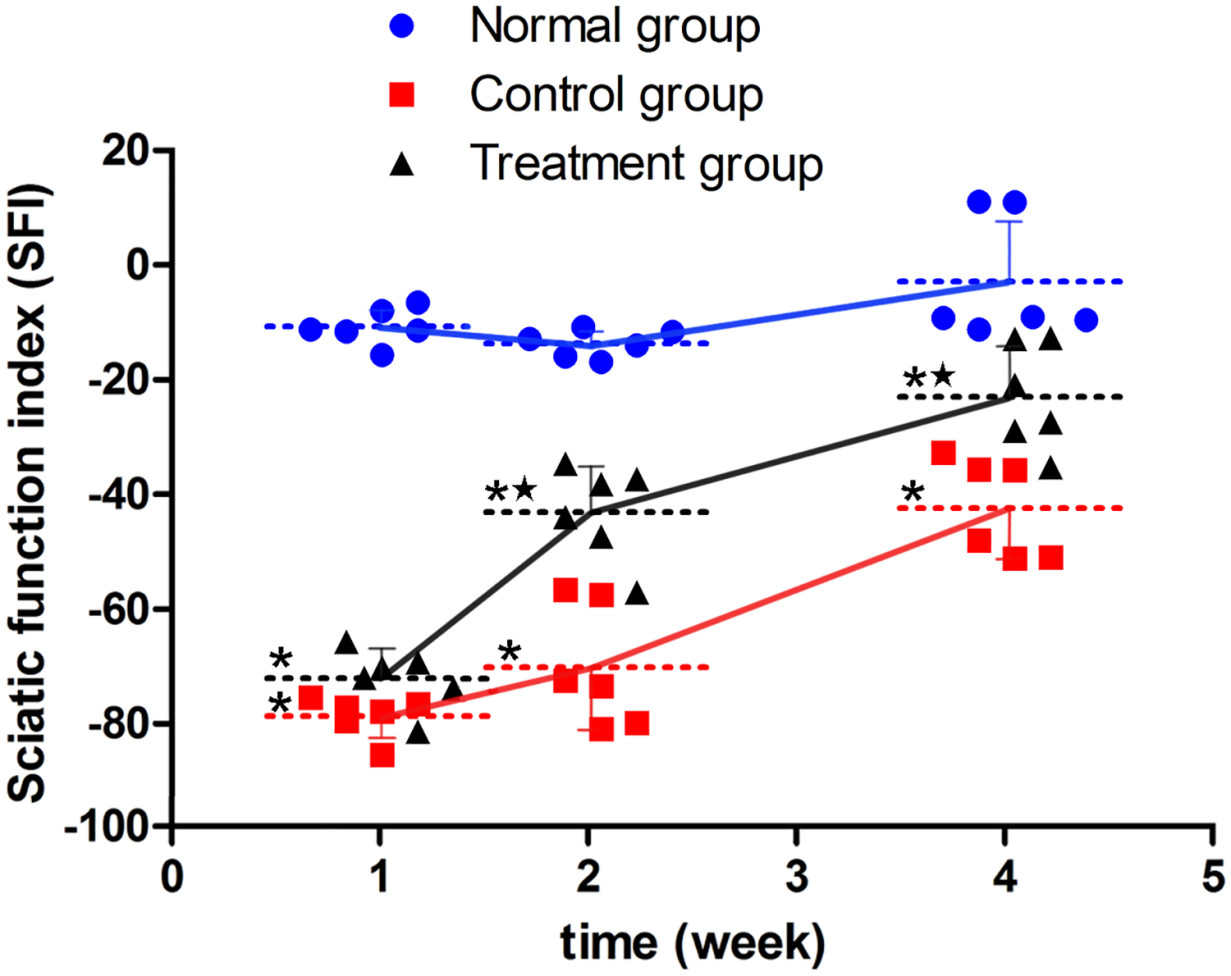
Motor function assessment. *P < 0.05 versus sham group; ^★^P < 0.05 versus control group. The error bars represent the standard deviation of SFI for each group (n = 6).

SFI values in the surgical groups were significantly lower than in the sham group 1 week after surgery (P < 0.05), while SFI values did not significantly differ between the control and treatment groups at this time. Two and 4 weeks after surgery, SFI values in the surgical groups were significantly lower than in the sham group (P < 0.05), and the values in the treatment group were significantly higher than in the control group (P < 0.05). SFI variation in the sham group was minimal over time. During the period from the first week to the fourth week after surgery, SFI values in the surgical groups gradually increased, and the range of variation from the first week to the second week was larger in the treatment group than in the control group. (Fig 2).

### 3.2. Mass and morphology of the tibialis anterior muscle

**Fig 3:**
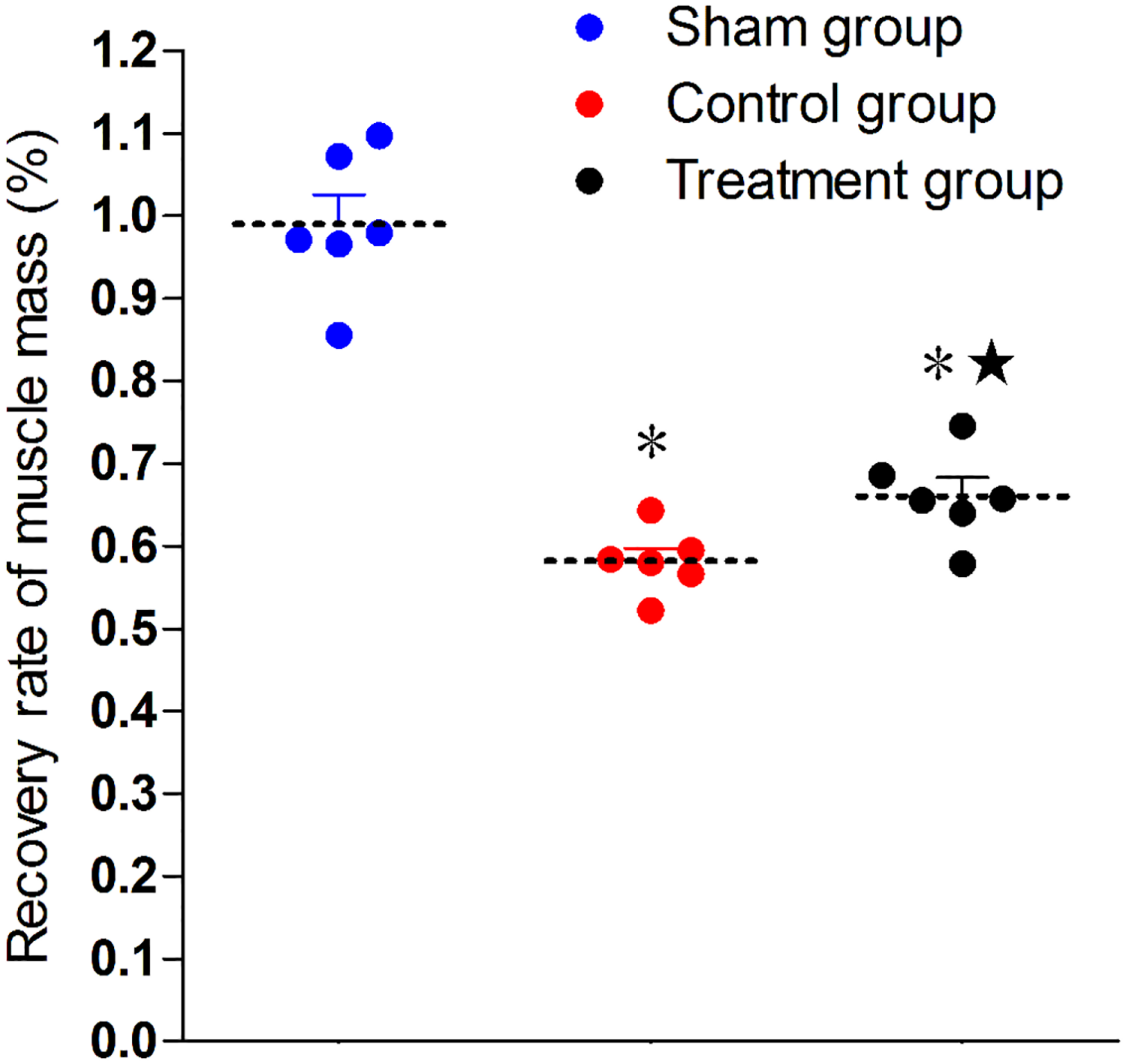
Recovery of anterior tibial muscle mass. *P < 0.05 versus sham group; ^★^P < 0.05 versus control group. The error bars represent the standard deviation of muscle mass recovery rate for each group (n = 6).

Four weeks postoperative, the weight of bilateral anterior tibialis muscle was determined. The weight ratios in the surgical groups were significantly lower than in the sham group, whereas the ratio was higher in the treatment group than in the control group (Fig 3).

**Fig 4:**
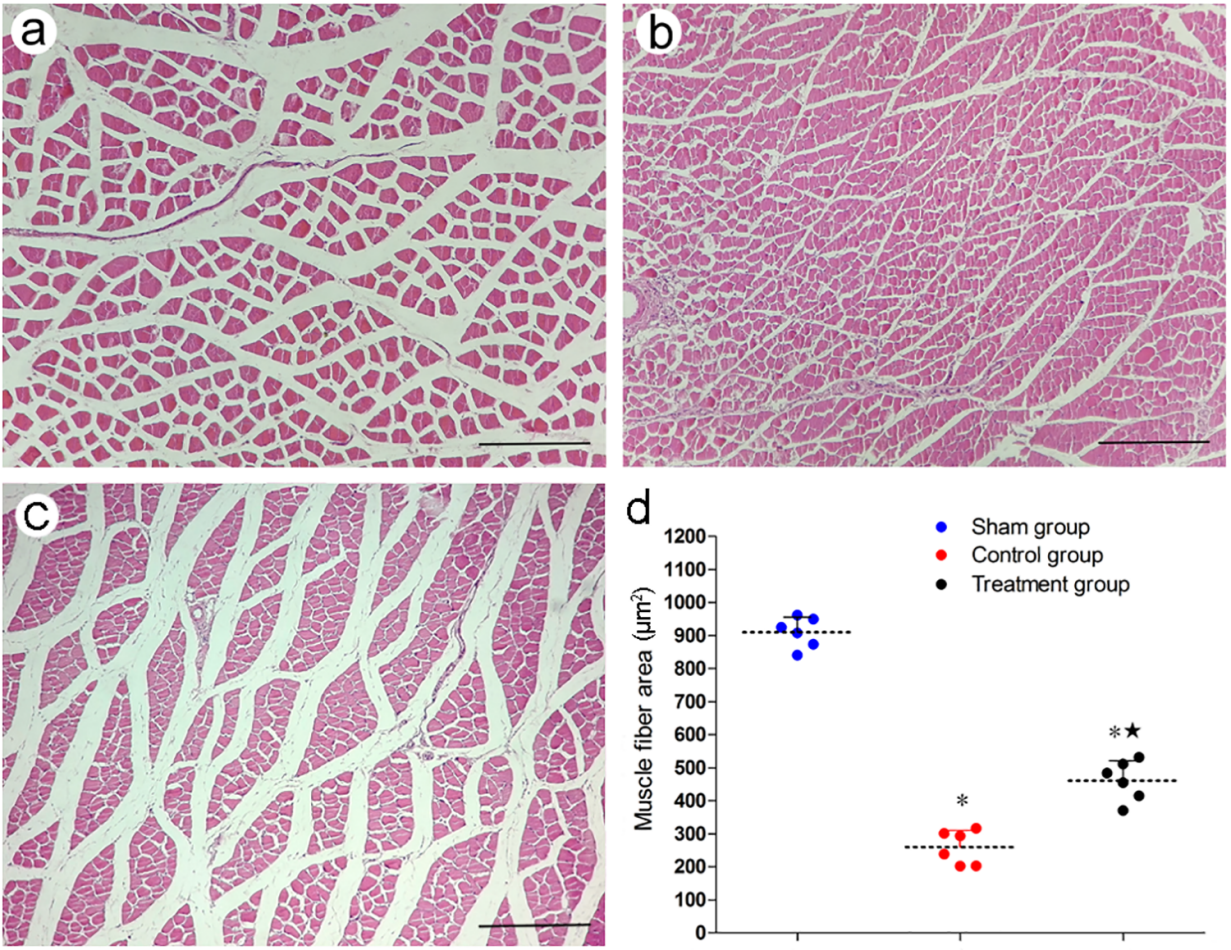
Morphological assessment of the tibialis anterior muscle. (a-c) Morphology of tibialis anterior muscle by H-E staining; (a) Sham group; (b) Control group; (c) Treatment group. Original magnification ×100; Scale bar = 200 μm. (d) Muscle fiber area analysis. *P < 0.05 versus sham group; ^★^P < 0.05 versus control group. The error bars represent the standard deviation of muscle fiber area for each group (n = 6).

The size of the ipsilateral tibialis anterior muscle decreased in the surgical groups 4 weeks after surgery, the histological appearance of the tibialis anterior muscles were significantly enhanced by Tanshinone IIA (Fig 4a–4c). Based on statistical results, the muscle fiber areas in the surgical groups were significantly lower than in the sham group (P < 0.05), while the area in the treatment group was significantly higher than in the control group (P < 0.05) (Fig 4d).

### 3.3. Quantity and morphometric measurements of myelinated fibers

**Fig 5:**
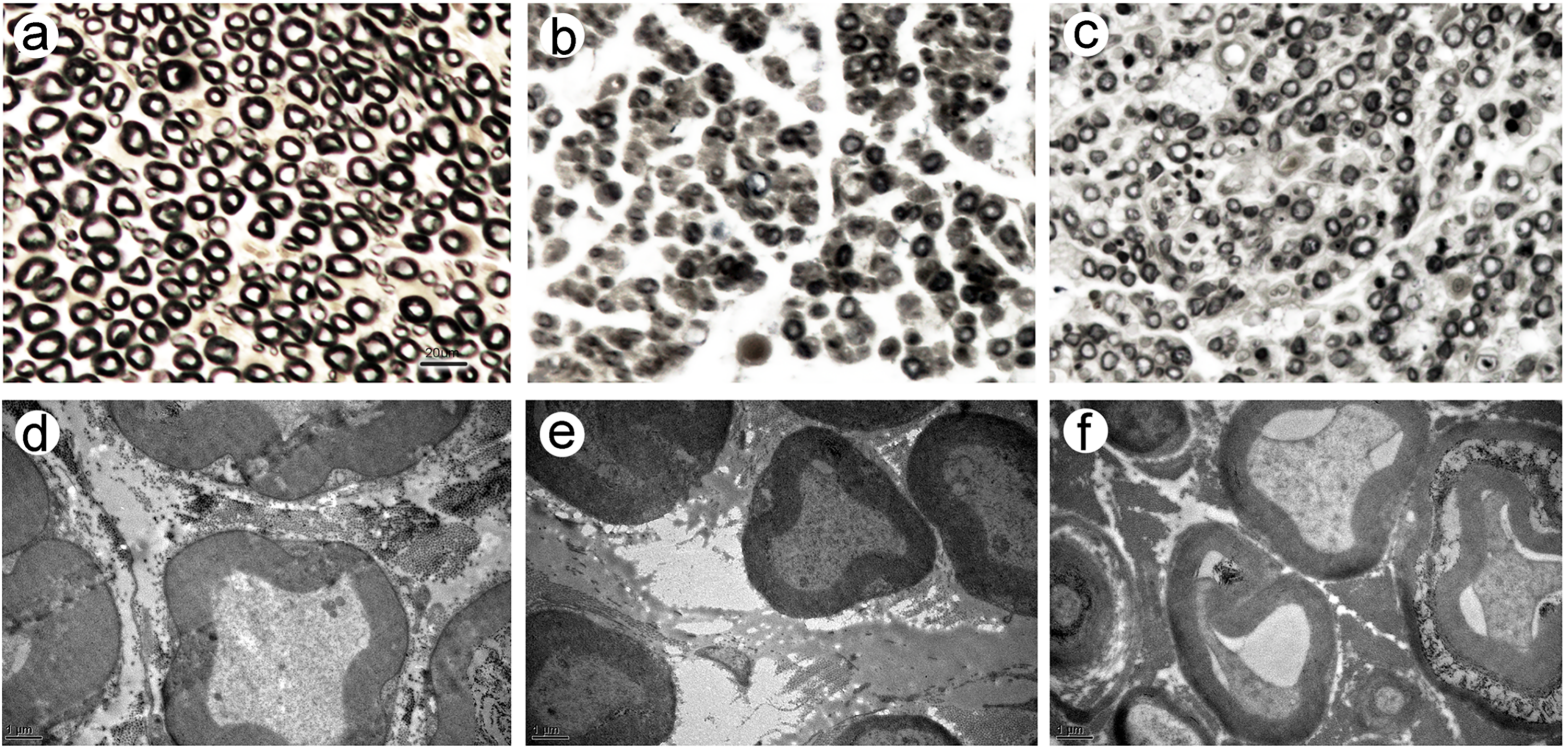
Morphology of myelinated fibers. (a-c) Osmium tetroxide staining; Original magnification ×400; Scale bar = 20 μm. (d-f) Myelinated fibers by TEM; Original magnification ×12000; Scale bar = 1 μm. (a) and (d) Sham group; (b) and (e) Control group; (c) and (f) Treatment group.

The density and diameter of myelinated fibers appeared smaller in the control group. Myelin regeneration looked relatively poor, and the distribution looked uneven. In the treatment group, the myelin density appeared higher (Fig 5).

The number of myelinated fibers per high power field (×400) was counted. The number of fibers in the sham group was higher than in the surgical groups (P < 0.05), whereas the number of fibers in the treatment group was higher than in the control group (P < 0.05). (see Table 1)

**Table 1:** The number and morphometric evaluation of myelinated fibers.

	Group		
	Sham group	Control group	Treatment group
Number of fibers	508.25±59.56	225.74±45.08*	327.63±53.62*^▲^
Fiber diameter (μm)	7.21±0.84	5.00±0.56*	5.42±0.51*^▲^
Axon diameter (μm)	3.33±0.55	2.01±0.51*	2.30±0.77*^▲^
G-ratio (Axon diameter/Fiber diameter)	0.44±0.13	0.41±0.10	0.42±0.15
Myelin thickness (μm)	1.94±0.44	1.49±0.32*	1.56±0.48*

*P < 0.05 versus sham group;

^▲^*P* < 0.05 versus control group

Myelin thickness in the sham group was significantly larger than those in the surgical groups (P < 0.05), whereas the values of the control and treatment groups did not significantly differ. Fiber and axon diameters were significantly larger in the sham group than in the surgical groups (P < 0.05), and the both diameters in the treatment group were significantly larger than in the control group (P < 0.05). (see Table 1)

### 3.4. Assessment of spinal cord neurons

**Fig 6:**
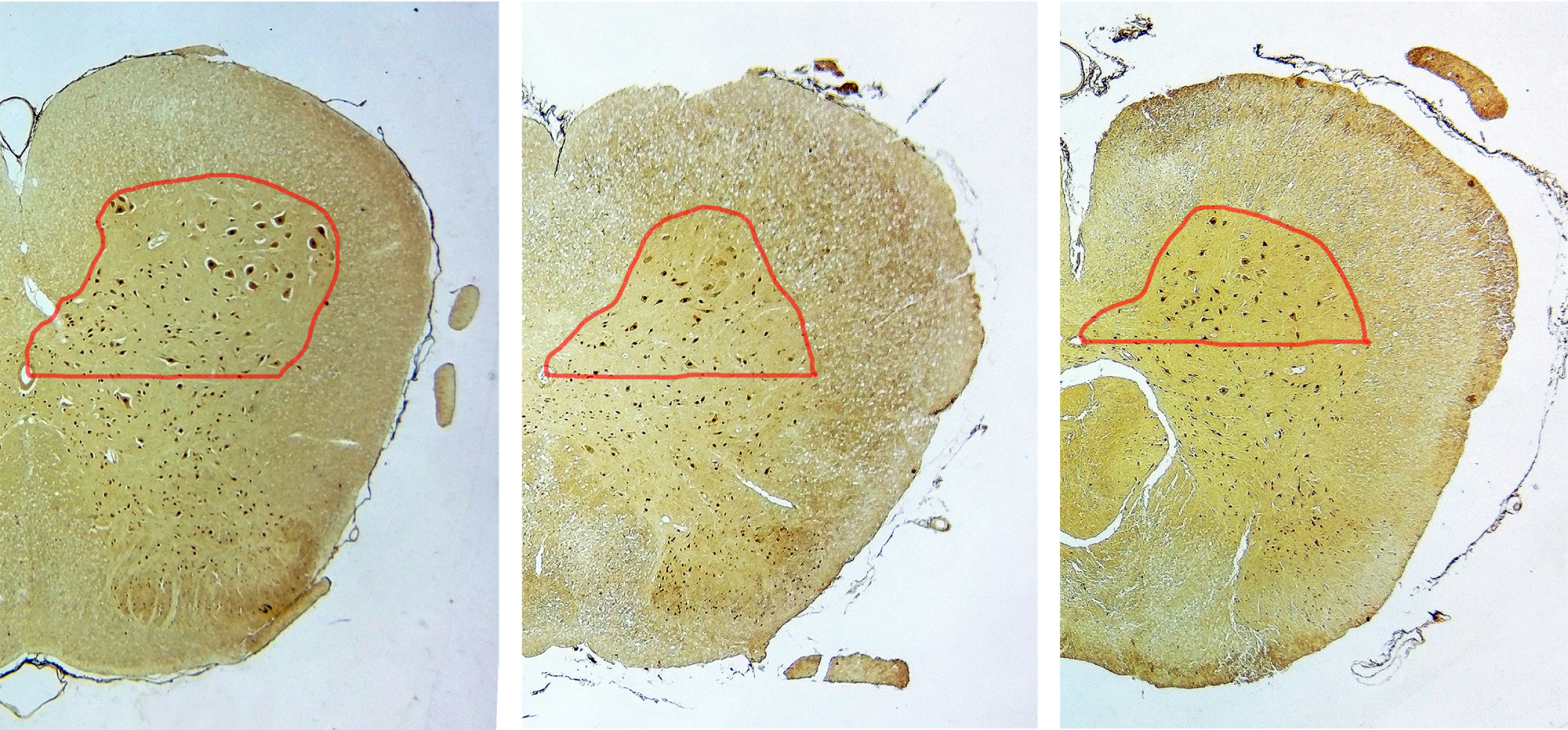
Motor neuron morphology of lumbar spinal cord. (a) Sham group; (b) Control group; (c) Treatment group. Original magnification ×40; Scale bar = 200 μm. Red curve indicates spinal ventral horn.

**Fig 7:**
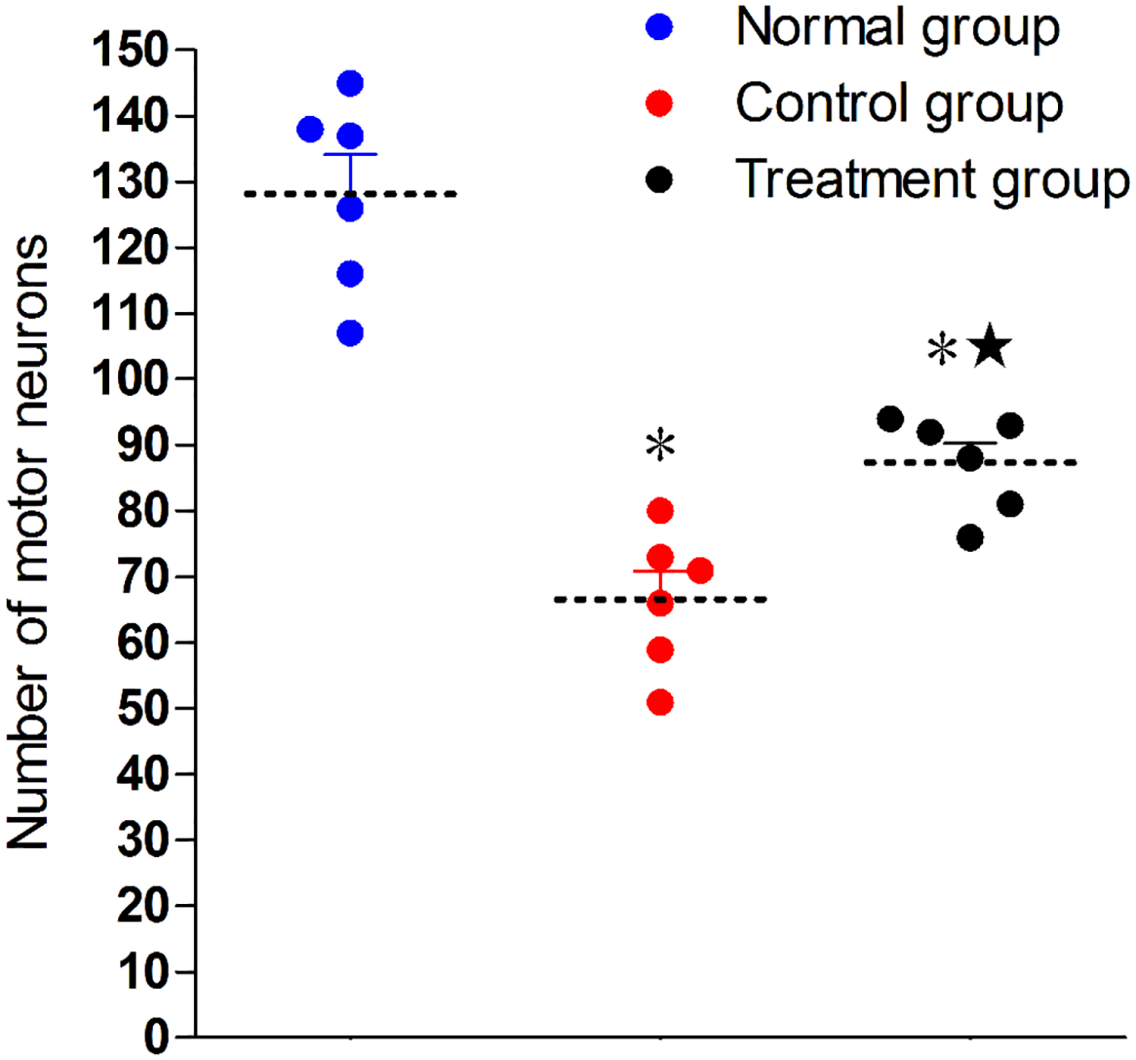
Number of motor neurons. *P < 0.05 versus sham group; ^★^P < 0.05 versus control group. The error bars represent the standard deviation of number of motor neurons for each group (n = 6).

The number of the motor neurons was higher in the sham group than in the surgical groups (P < 0.05). The treatment group showed a much higher number of motor neurons than the control group (P < 0.05). (Figs 6 and 7)

## 4. Discussion

After peripheral nerve crush injury, nerve fibers are damaged and transected because of mechanical compression exerted by the clamp, whereas the epineurial and endoneurial tubes remain intact, as in a typical crush injury. Nerve fibers distal to the injury site undergo Wallerian degeneration [9], distal axons and myelin sheaths degenerate. Büngner tubes are formed by proliferated Schwann cells to guide the proximal axial buds to regenerate. In addition, several neurotrophic factors secreted from Schwann cells and target organs induce the proximal axons to extend into the distal endoneurial tubes [10]. However, nerve regeneration is generally insufficient for functional recovery. It is necessary to use adjuvant therapy in nerve crush injury model.

Chinese herbal medicine is often used as adjuvant therapy for nerve injury because of positive effect and few side effects. However, Chinese herbal medicines that more effectively treat nerve crush injury have yet to be discovered. Thus, there is a need for further exploration to identify effective Chinese herbs.

In an effort to facilitate nerve repair, we evaluated the feasibility of Tanshinone IIA as an adjuvant treatment drug to promote regrowth after nerve injury. Tanshinone IIA is a primary component of Danshen, a popular traditional Chinese medicine. Tanshinone IIA has been proved to attenuate ischemic injury and to play positive role in cerebrovascular disease therapy [11]. More importantly, Tanshinone IIA prevent the brain from transient focal cerebral ischemia and neonatal hypoxic-ischemic injury [12], indicating a positive effect on neuroprotection. It is noteworthy that, according to a previous study, Tanshinone IIA attenuated injury after sciatic nerve transection [6], suggesting the therapeutic effect of this drug on peripheral nerve injury. In addition to the above-mentioned sciatic nerve disconnection model, the effect of Tanshinone IIA on the sciatic nerve clamp injury model is expected to be positive, being worthy of being verified in this study.

The results in this study suggested that Tanshinone IIA attenuated damage of neural structure and function. Administration of Tanshinone IIA improved motor function, indicated by increased SFI and increased recovery rate of muscle mass. Tanshinone IIA also improved the nerve structure by increasing the fiber and axon diameters as well as the quantity of myelinated fibers. Tanshinone IIA achieved better nerve regeneration outcomes than saline, demonstrating its potential therapeutic effect on peripheral nerve injury.

In addition, a larger number of motor neurons was observed in the rats being given adjuvant treatment compared with the rats without treatment, suggesting that Tanshinone IIA reduces neuronal death. The mechanisms behind its effect on neuronal death are likely complex. According to earlier reports, Tanshinone IIA has antioxidant activity and can mitigate apoptotic pathways [13], preventing oxidative stress that induces the apoptotic death of neurons [14]. In addition, Tanshinone IIA can increase fibroblast growth factor-2 that aids nerve regeneration [15]. Tanshinone IIA also increase the proliferation along with migration of Schwann cells via extracellular signal-regulated kinase as well as NH2-terminal kinase signaling pathways [16]. In this study, Tanshinone IIA probably attenuated nerve structural and functional damage induced by nerve crush injury through the above mechanisms, though the exact mechanisms remain to be determined.

The results of this study demonstrated the positive efficacy and few side effects of Tanshinone IIA in treating sciatic nerve crush injury in Sprague Dawley rats. In addition, Tanshinone IIA can be injected daily and is relatively inexpensive compared with other drug therapies such as neurotrophic factors.

In the present study, only motor function recovery was explored, however, normal neurological functions include both motor and sensory components. Based on the conclusion that Tanshinone IIA plays a positive role in neural structure and motor function, its effect on sensory functions, such as static mechanical allodynia, dynamic mechanical allodynia, heat hyperalgesia, mechanical hyperalgesia, and cold allodynia, warrants investigation in future studies.

## 5. Conclusions

Tanshinone IIA attenuates nerve structural and functional damage induced by nerve crush injury in this exploratory study of a sciatic nerve injury rat model.

## Supporting information

S1 File Numerical values of Fig 2. (XLSX)

S2 File Numerical values of Fig 3. (XLSX)

S3 File Numerical values of Fig 4. (XLSX)

S4 File Numerical values of Fig 7. (XLSX)
